# Glutathione-depleting self-immolative nanoparticles boost cuproptosis-driven metalloimmunotherapy for triple-negative breast cancer

**DOI:** 10.1186/s12951-026-04668-9

**Published:** 2026-07-07

**Authors:** Yunhai Fu, Xiaoyu Wang, Yinqi Li, Xiaodong Yang, Ziying Yao, Jingwen Wang, Yaxin Zhang, Tianping Li, Yunke Zhu, Zhiyao He

**Affiliations:** 1 Department of Pharmacy, Cancer Center and State Key Laboratory of Biotherapy, West China Hospital, Sichuan University, Chengdu, 610041 China; 2 Key Laboratory of Drug-Targeting and Drug Delivery System of the Education Ministry and Sichuan Province, Sichuan Engineering Laboratory for Plant-Sourced Drug and Sichuan Research Center for Drug Precision Industrial Technology, School of Pharmacy, Sichuan University, West, Chengdu, 610041 China; 3 Institute of Thoracic Oncology and Department of Thoracic Surgery, West China Hospital, Sichuan University, Chengdu, 610041 China; 4 Department of Thoracic Surgery, Chengdu Shangjin Nanfu Hospital, Chengdu, 611730 China

**Keywords:** Triple-negative breast cancer, Enhanced cuproptosis, Tumor immune microenvironment reprogramming, Neoadjuvant potential, Metalloimmunotherapy

## Abstract

Despite advances in immunotherapy, triple-negative breast cancer (TNBC) remains a major therapeutic challenge owing to its marked aggressiveness, low immunogenicity, and limited responsiveness to immune checkpoint blockade (ICB). Herein, the esterase-responsive polymeric nanoparticles (EsCu@NPs) are developed. The polymer backbone covalently integrates an L-buthionine sulfoximine prodrug to inhibit glutathione biosynthesis, and the nanoparticle matrix encapsulates an elesclomol-copper complex to promote intratumoral copper accumulation. By concurrently depleting glutathione and perturbing copper homeostasis, EsCu@NPs elicit reinforced cuproptosis stress that is converted into immunogenic cell death. This process reprograms the suppressive tumor microenvironment, characterized by increased DC maturation, elevated CD8⁺ T-cell infiltration, and a reduction in regulatory T cells. These findings support enhanced cuproptosis-driven metalloimmunotherapy as a strategy for treating TNBC. Briefly, in neoadjuvant settings, preoperative EsCu@NPs reduce tumor burden, decrease minimal residual disease, curb postsurgical recurrence and spontaneous lung metastasis, translating into prolonged survival. Notably, combination with anti-PD-1 checkpoint blockade yields synergy, significantly enhancing the ICB responsiveness of TNBC and producing improved therapeutic efficacy. Overall, this study demonstrates that EsCu@NPs enable coordinated cuproptosis and immune reprogramming, expanding ICB efficacy and supporting a translatable neoadjuvant strategy in TNBC.

**Graphical Abstract:**

**Figure:**
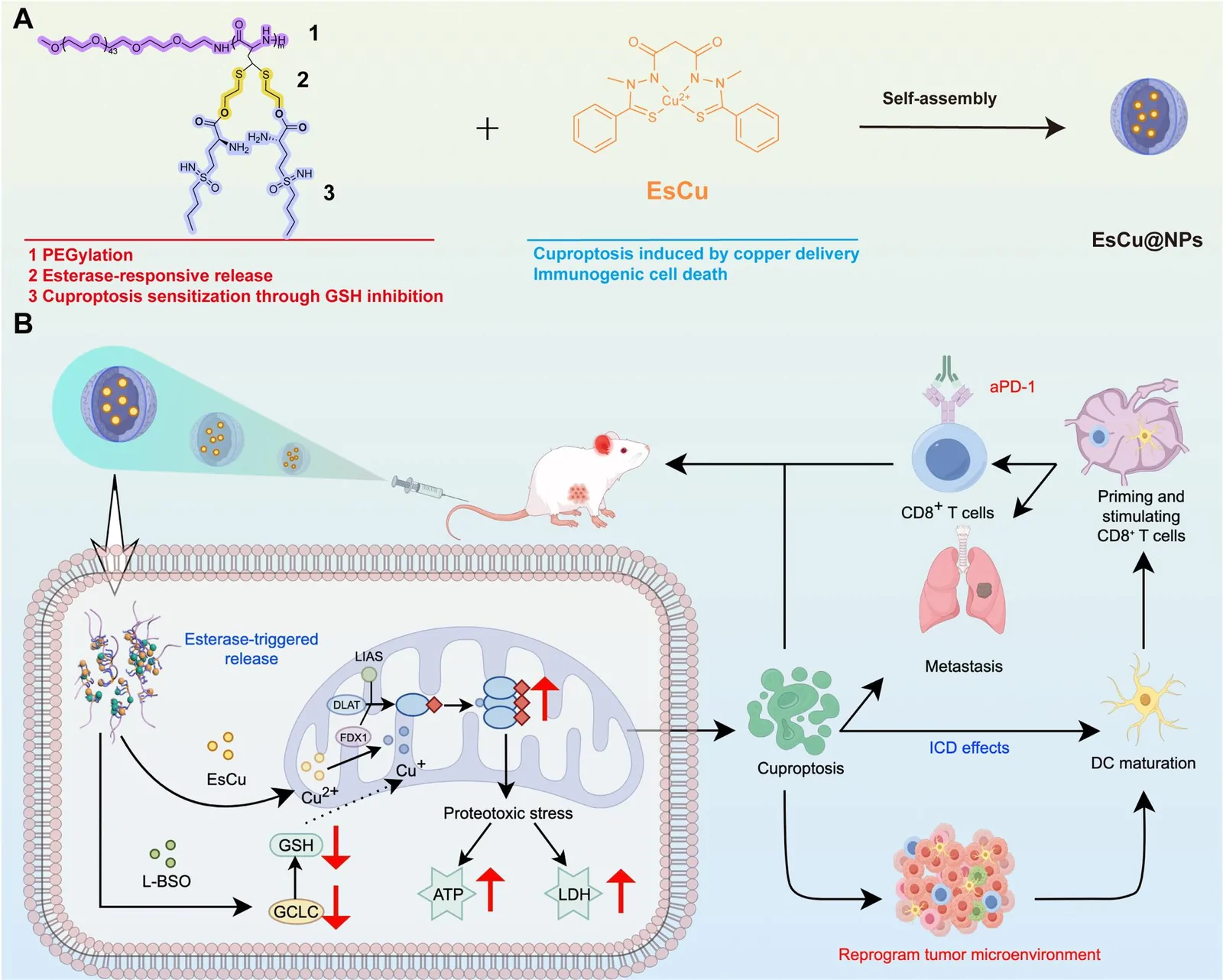

**Supplementary Information:**

The online version contains supplementary material available at https://doi.org/10.1186/s12951-026-04668-9.

## Introduction

Immune checkpoint blockade (ICB) has changed treatment in several malignancies by mobilizing the host immune system to eradicate cancer cells [1–5]. However, in triple-negative breast cancer (TNBC), clinical benefit remains heterogeneous and often non-durable [6], largely due to low tumor immunogenicity and a profoundly immunosuppressive tumor immune microenvironment (TIME) [7–9]. Moreover, reliance on biomarker variability [7], context-dependent responses, and the risk of immune-related adverse events further complicate its widespread application [10, 11]. These limitations underscore the need for mechanism-informed priming strategies that can convert immunologically cold TNBC into an ICB-permissive state before definitive locoregional therapy [12–14].

In recent years, metalloimmunotherapy, an emerging strategy that harnesses metal ions as immune-modulatory effectors, has shown distinctive promise in oncology [15–17]. Among these approaches [18–20], cuproptosis has been identified as a mitochondria-dependent, regulated cell death process triggered by copper overload [21–23]. Excess copper binds directly to lipoylated tricarboxylic acid cycle proteins, provoking proteotoxic stress through mitochondrial regulators such as ferredoxin 1 (FDX1) [24]. Unlike conventional cytotoxicity, appropriately tuned copper stress not only kills tumor cells but also remodels the immunosuppressive TIME by inducing immunogenic cell death (ICD) [25–27], enhancing antigen presentation, and promoting T-cell infiltration, thereby providing a metabolic entry point to convert cold tumors into hot ones [28]. Building on this foundation, cuproptosis-driven metalloimmunotherapy offers a new horizon for TNBC [29]. More importantly, molecular evidence supports this concept: genes involved in cuproptosis show marked dysregulation and prognostic relevance in breast cancer [30]. For example, solute carrier family 31 member 1 (SLC31A1) is upregulated in triple-negative breast cancer and correlates with poor prognosis [31]; conversely, cuproptosis-related gene scores indicate that patients with higher scores have worse overall survival yet display greater sensitivity to ICB. In contrast, high expression of lipoic acid synthetase (LIAS) is associated with better overall survival, distant metastasis-free survival, progression-free survival, and recurrence-free survival [32]. These associations underscore the biological heterogeneity and context dependence of the cuproptosis pathway: it is closely linked to tumor progression, yet appropriate copper homeostasis intervention may increase sensitivity to immunotherapy. Together, these insights provide a theoretical foundation for a metabolism-immune co-engineering strategy in TNBC.

However, translating cuproptosis into an effective therapeutic approach for TNBC faces several challenges [29]. Small-molecule ionophores often fail to achieve tumor-specific copper delivery and display unstable pharmacokinetics [20]. The glutathione (GSH)-rich cytosol of TNBC reduces Cu²⁺ to Cu⁺, quenches oxidative stress, and protects lipoylated mitochondrial proteins, thereby dampening protein aggregation and cuproptosis-associated proteotoxic stress [33]. Even when cytotoxicity occurs, the resulting signals frequently fail to convert the TIME into a T-cell-permissive state, limiting synergy with ICB. Several copper-delivery strategies have been developed, such as inorganic copper nanocrystals [34], copper-based metal-organic frameworks [35], and polymeric carriers of copper complexes [36]. Despite their diversity, these systems share similar limitations, including copper leakage, off-target toxicity, insufficient tumor selectivity, and rapid clearance by the reticuloendothelial system [20, 37]. Furthermore, most fail to counteract elevated intratumoral GSH or induce robust immune activation, making durable T-cell-mediated tumor control difficult to achieve [38].

To address these barriers, we designed an esterase-responsive polymeric nanoplatform (termed EsCu@NPs) whose polymer backbone covalently integrates the L-buthionine sulfoximine (L-BSO) prodrug (an inhibitor of GSH biosynthesis) [39], while the particle matrix encapsulates the elesclomol-copper complex. This dual architecture is intended to amplify cuproptosis and trigger metal-mediated immunoactivation, ultimately remodeling the TIME to suppress TNBC progression, recurrence, and metastasis. Functionally, the platform serves as a copper vector, enabling efficient intratumoral copper accumulation and cuproptosis for direct tumor killing. Tumor-overexpressed esterases cleave the prodrug linker in the backbone to release L-BSO, which inhibits key enzymes in GSH biosynthesis, reduces endogenous GSH levels, removes redox defenses, and sensitizes tumors to copper-induced stress. By coupling mitochondrial copper delivery with GSH blockade, EsCu@NPs intensify cuproptosis and induce ICD, thereby promoting dendritic cell maturation, cross-presentation, and CD8⁺ T-cell infiltration **(**Scheme 1**)**. In parallel, EsCu@NPs alleviate T-cell exhaustion, collectively preheating tumors and rendering them more responsive to anti–programmed cell death protein 1 antibody (aPD-1). EsCu@NPs, when used as neoadjuvant therapy, can reduce the risk of postoperative metastasis and recurrence in TNBC, while simultaneously remodeling the TIME and enhancing sensitivity to ICB, thereby offering greater potential for combination immunotherapy. In turn, aPD-1 releases inhibitory brakes on T cells and strengthens effector functions, creating a bidirectional synergy that may yield more pronounced and durable therapeutic effects. Collectively, this strategy defines a translational cuproptosis-immune co-engineering pathway; when combined with aPD-1, it is expected to achieve broader, more potent, and longer-lasting clinical benefits.

**Fig. 1:**
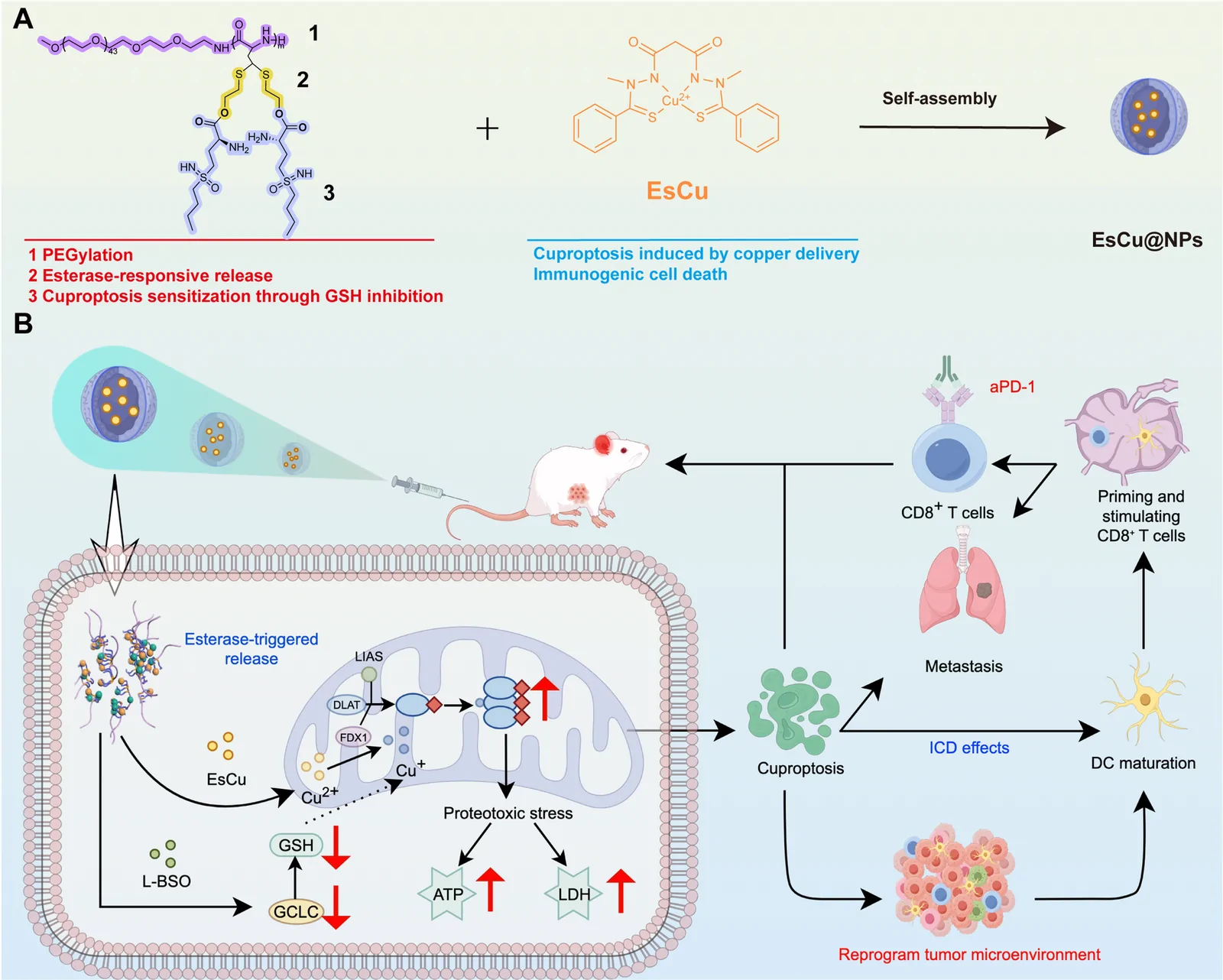
Construction of a stimuli-responsive polymeric nanocarrier that induces cuproptosis for synergistic immunotherapy. (**A**) Self-assembly of EsCu@NPs from L-BSO-conjugated polymer and EsCu. (**B**) EsCu@NPs promote copper accumulation and GSH depletion to induce cuproptosis, activate antitumor immunity, inhibit metastasis, and enhance anti-PD-1 therapy

## Materials and methods

### Main materials and animals

Unless otherwise indicated, all chemicals were purchased from Bide Pharmatech Ltd. (Shanghai, China). The LysoTracker Green (catalog no. C1047S), BCA protein assay kit (catalog no. P0012S), RIPA lysis buffer (catalog no. P0013B) and ATP assay kit (catalog no. S0026) were purchased from Beyotime Biotechnology Co., Ltd. (Shanghai, China). The GSH assay kit (catalog no. BC1175) was obtained from Solarbio Science & Technology Co., Ltd. (Beijing, China). The Annexin V-FITC (catalog no. 40302ES20) was obtained from Yeasen Biotechnology Co., Ltd. (Shanghai, China). Anti-mouse FDX1 (catalog no. 12592-1-AP), DLAT (catalog no. 13426-1-AP), GCLC (catalog no. 12601-1-AP), and LIAS (catalog no. 11577-1-AP) antibodies were sourced from Proteintech Biotechnology Co., Ltd. (Wuhan, Hubei, China). The lactate dehydrogenase (LDH) assay kit (catalog no. E-BC-K046-M) was supplied by Elabscience Biotechnology Co., Ltd. (Wuhan, Hubei, China). Enzyme-linked immunosorbent assay kits for TNF-α (catalog no. 88-7324-88), IL-4 (catalog no. BMS613), and IFN-γ (catalog no. 88-8314-88) were obtained from Thermo Fisher Scientific Co., Ltd. (Waltham, Massachusetts, USA). The aPD-1 (catalog no. HRP00262-023) was provided by Hengrui Medicine Co., Ltd. (Lianyungang, Jiangsu, China). All cells were cultured at 37 °C in a humidified incubator with 5% CO_2_. BALB/c mice (female, 16–18 g, 6–8 weeks old) were bought from Beijing Vital River Laboratory Animal Technology Co., Ltd. (Beijing, China).

### Preparation of EsCu@NPs

The ELPM polymer was dissolved in dimethyl sulfoxide (10 mg/mL). Es and CuCl_2_ were also dissolved in dimethyl sulfoxide (at a molar ratio of Es to Cu²⁺ of 1:1). Subsequently, 100 µL of the ELPM solution was dispersed into deionized water, and 10 µL of the EsCu solution (Es 100 µg/mL) was added to the water, followed by 5 min of sonication to obtain EsCu@NPs. The morphology and elemental composition of the nanoparticles were examined using transmission electron microscopy (JEM-2100Plus, JEOL, Tokyo, Japan). The hydrodynamic size distribution and zeta potential of the nanoparticles were analyzed using a dynamic light scattering analyzer (Zetasizer Nano, Malvern, Worcestershire, UK). The copper ion content was determined by inductively coupled plasma mass spectrometry (ICP, Agilent, Santa Clara, California, USA). The release kinetics of EsCu were detected by HPLC (LC-2050 C, Shimadzu, Tokyo, Japan).

### In vitro cellular uptake

The 4T1 cells were seeded in 6-well plates and incubated overnight. Cells were then incubated with indocyanine green (ICG) and ICG@NPs at the same ICG concentration (8 µg/mL) for various time intervals. Blank 4T1 cells without ICG or ICG@NPs were used as negative controls.The intracellular fluorescence intensity of ICG was measured by flow cytometry (BD FACS Fortessa, BD Biosciences, Franklin Lakes, New Jersey, USA). For confocal laser scanning microscopy (LSM 980, Carl Zeiss, Jena, Germany) analysis, pre-seeded 4T1 cells were incubated with ICG@NPs and ICG at the same concentration for 24 h. After incubation, the cells were washed with PBS and stained with LysoTracker Green for 50 min for labeling lysosomes and DAPI for labeling nuclei.

### Measurement of intracellular GSH levels

The 4T1 cells were seeded at a density of 5 × 10^5^ cells per well in 6-well plates and incubated at 37 °C in a humidified incubator containing 5% CO_2_ for 12 h. Then, 4T1 cells were treated for 24 h with control, Es, CuCl_2_, EsCu, Free@NPs, Es@NPs, and EsCu@NPs. The concentrations of Es and CuCl_2_ in the respective groups were 50 nM. After treatment, cells were collected and washed twice with PBS. The GSH content in each group was determined using a commercial GSH assay kit according to the manufacturer’s instructions.

### Cytotoxicity assay

MCF-7 and 4T1 cells were seeded in 96-well plates at a density of 5,000 cells per well and incubated overnight at 37 °C under 5% CO_2_. Subsequently, the culture medium was replaced with fresh medium containing Es, CuCl_2_, EsCu, Free@NPs, Es@NPs and EsCu@NPs at various concentrations. After an additional 24 h incubation, 10 µL of MTT solution was added to each well. Following a further 4 h incubation, the absorbance at 560 nm was measured using a microplate reader to evaluate cell viability.

### Apoptosis analysis by flow cytometry

The 4T1 cells were seeded in 6-well plates and incubated overnight. Then, 4T1 cells were treated for 24 h with control, Es, CuCl_2_, EsCu, Free@NPs, Es@NPs, and EsCu@NPs. The concentrations of Es and CuCl_2_ in the respective groups were 50 nM. After treatment, cells were washed three times with PBS, detached with trypsin, collected by centrifugation, and resuspended in PBS. Cells were then stained with Annexin V-FITC and propidium iodide, followed by flow cytometric analysis of apoptosis.

### Western blot

The 4T1 cells were seeded at a density of 3 × 10^5^ cells per well in 6-well plates and incubated at 37 °C in a humidified incubator containing 5% CO_2_ for 12 h. Then, 4T1 cells were treated with control, Es, CuCl_2_, EsCu, Free@NPs, Es@NPs, and EsCu@NPs. The concentrations of Es and CuCl_2_ in the respective groups were 50 nM. The 4T1 cells were collected after the specified time points, homogenized in RIPA lysis buffer and centrifuged (12,000 rpm, 10 min) to obtain protein solutions. The protein concentration was measured using a BCA protein assay kit. The samples (20 µg total protein) were separated on SDS polyacrylamide gels. The separated protein bands were transferred onto a PVDF membrane (220 mA, 2 h). The PVDF membranes were soaked in 5% BSA for 2 h and then washed with TBST. Next, the PVDF membranes were incubated with primary antibodies FDX1 (1:2000), LIAS (1:2500), DLAT (1:5000), GCLC (1:5000), TXNRD1 (1:2000) and GAPDH (1:50000) at 4 °C for 12 h and washed with TBST. Then, the PVDF membranes were incubated with a secondary antibody (1:50,000) for 1 h and washed with TBST. Finally, the PVDF membranes were detected using an eBLOT touch imager (eBLOT, Hangzhou, Zhejiang, China).

### Induction of ICD with EsCu@NPs

4T1 cells were seeded in glass-bottom confocal dishes and cultured for 12 h. The cells were treated with control, Es, CuCl_2_, EsCu, Free@NPs, Es@NPs, and EsCu@NPs for 8 h. The cells were washed with PBS and fixed with 4% paraformaldehyde for 10 min. After blocking the cells for 1 h, anti-CRT and anti-HMGB1 antibodies were added and incubated at 4 °C overnight. Finally, the cells were further incubated with secondary antibody for observation by inverted fluorescence microscope, and the nuclei of 4T1 cells were stained with Hoechst 33,342.

To determine nanocarrier-induced ICD, LDH release and ATP secretion were examined. Briefly, 4T1 cells (1 × 10^5^ cells per well) were seeded on 12-well plates and incubated for 24 h. The cells were treated with control, Es, CuCl_2_, EsCu, Free@NPs, Es@NPs, and EsCu@NPs for 12 h. The concentrations of Es and CuCl_2_ in the respective groups were 50 nM. The cell culture supernatant was collected, and the sample was placed on ice. The ATP concentration was detected using the ATP assay kit. The LDH concentration was detected using the LDH assay kit.

Bone marrow cells were isolated from the femurs and tibias of mice and cultured in RPMI-1640 medium supplemented with 10% FBS, 20 ng/mL recombinant mouse GM-CSF, and 10 ng/mL recombinant mouse IL-4 to induce differentiation into bone marrow-derived dendritic cells (BMDCs). 4T1 cells were treated with control, Es, CuCl_2_, EsCu, Free@NPs, Es@NPs, and EsCu@NPs for 12 h. The conditioned medium was collected by centrifugation at 3,000 rpm for 5 min to remove cell debris and then added to immature BMDCs. After incubation for 24 h, BMDCs were collected and analyzed by flow cytometry. Mature BMDCs were defined as CD11c⁺CD80⁺CD86⁺ cells.

### Animal models

To establish the 4T1 subcutaneous tumor model, 4T1 cells (1 × 10^6^) suspended in 100 µL of RPMI-1640 medium was injected into the right inguinal region of BALB/c mice. To establish the 4T1 orthotopic mammary tumor model, 4T1-Luc cells (1 × 10^6^) suspended in 100 µL of RPMI-1640 medium was injected into the mammary fat pad of BALB/c mice. Tumor volume was calculated using the formula: volume = 0.5 × (tumor length) × (tumor width)^2^. Mice were euthanized when the tumor volume reached 1500 mm^3^.

### Analysis of peripheral and tumor-infiltrating immune cells

To study the immune responses, subcutaneous 4T1 tumor–bearing mice received three intravenous injections of the following formulations: control, Es, CuCl_2_ (1.8 mg/kg), EsCu, Free@NPs, Es@NPs, and EsCu@NPs (Es, 6 mg/kg). The spleens, lymph nodes, and tumors were collected for immune cell analysis. CD8⁺ T cells were isolated from the spleen and lymph nodes using a magnetic bead-based CD8⁺ T-cell isolation kit, followed by measurement of intracellular GSH levels in the purified CD8⁺ T-cell population.

For flow cytometric analysis of peripheral immune cells, spleens were harvested, mechanically dissociated, and passed through a 40 μm cell strainer to obtain single-cell suspensions. Red blood cells were lysed using red blood cell lysis buffer, and the remaining cells were washed with PBS. To analyze tumor-infiltrating lymphocytes, primary tumors were harvested to isolate lymphocytes. Tumor tissues were minced into small pieces and digested at 37 °C for 30 min in RPMI-1640 medium containing 1 mg/mL collagenase IV, 0.1 mg/mL hyaluronidase, 0.1 mg/mL DNase I, and 2% fetal bovine serum. The digested tissues were dissociated using a gentleMACS Dissociator, and single-cell suspensions were obtained by filtration through a 40 μm cell strainer. Cells were purified by Percoll gradient centrifugation and subsequently incubated on ice with Mouse TruStain FcX™ for 10 min to block Fc receptors. The single-cell suspensions were then stained with the following anti-mouse antibodies according to the manufacturer’s instructions: CD45, CD3, CD4, CD8, CD25, FOXP3, CD11c, CD80, CD86, PD-1, LAG-3, and TIM-3. Finally, the stained cells were analyzed using a flow cytometer (BD Biosciences, Franklin Lakes, New Jersey, USA), and data were processed with FlowJo software.

### Enzyme-linked immunosorbent assay

Following various treatments, tumor tissues were harvested and immediately placed on ice. The tissues were then homogenized and centrifuged to obtain the supernatants. Tumor biochemical indices and cytokine levels were measured using ELISA kits according to the manufacturer’s instructions.

### In vivo imaging

When the tumors reached around 100 mm^3^, 100 µL of ICG or ICG@NPs (containing ICG at 0.5 mg/mL) was injected intravenously. Then, the mice were imaged using in vivo imaging system (IVIS Lumina II, PerkinElmer, Shelton, Texas, USA) at 1, 12, 24, and 48 h post–drug injection.

### In vivo pharmacokinetic profile of EsCu@NPs

Mice were randomly divided into two groups and intravenously injected with EsCu or EsCu@NPs at an equivalent Es dose of 6 mg/kg. Blood samples were collected at predetermined time points of 1, 5, 30, 60, 360, 720, and 1440 min after injection. The collected blood samples were centrifuged to obtain plasma, and the concentration of EsCu was determined by HPLC.

### In vivo antitumor efficacy of subcutaneous tumor model

When the subcutaneous breast tumor volume reached approximately 100 mm^3^, BALB/c mice were randomly divided into 7 groups and administered different samples via tail vein injection four times (on days 0, 3, 6, 9), including control, Es, CuCl_2_ (1.8 mg/kg), EsCu, Free@NPs, Es@NPs, and EsCu@NPs (Es 6 mg/kg). Tumor volumes and body weights of each mouse were measured every other day. Mice were euthanized when the tumors in the control group reached a volume of approximately 1500 mm^3^. Tumor tissues were then harvested for subsequent analyses.

### Antitumor efficacy of orthotopic tumors and the spontaneous metastasis

The 4T1-Luc cells (1 × 10^6^ cells per mouse) were injected orthotopically into the right mammary fat pad of the female BALB/c mice. When the orthotopic tumor grew to 100 mm^3^, we randomly divided the mice into 7 groups (*n* = 5), and the mice received the following treatments via tail vein injection: control, Es, CuCl_2_ (1.8 mg/kg), EsCu, Free@NPs, Es@NPs, and EsCu@NPs (Es 6 mg/kg). The volumes of orthotopic tumors and the body weight of all mice were recorded every other day. On day 9, the orthotopic 4T1-Luc tumor was surgically removed, and the spontaneous lung metastasis was detected using IVIS (IVIS Lumina II, PerkinElmer, Shelton, Texas, USA).

### Antitumor efficacy of the combination of EsCu@NPs and aPD-1

When the subcutaneous tumor volume reached approximately 100 mm^3^, BALB/c mice were randomly divided into 4 groups and the mice received the following treatments via tail vein injection: control, EsCu@NPs (Es, 6 mg/kg), aPD-1 (10 mg/kg), EsCu@NPs + aPD-1 (Es, 6 mg/kg; aPD-1 10 mg/kg). Tumor volumes and body weights of each mouse were measured every other day. Mice were euthanized when the tumors in the control group reached a volume of approximately 1500 mm^3^. Tumor tissues were then harvested for subsequent analyses.

### Safety evaluation

Blood was collected from female mice and transferred into anticoagulant tubes. Erythrocytes were isolated by centrifugation at 3,000 rpm for 5 min, washed three times with PBS, and diluted with PBS to a final concentration of 5% (v/v). The erythrocyte suspension was incubated with EsCu@NPs at different concentrations ranging from 0.0625 to 2 mg/mL. PBS and Triton X-100 were used as the negative and positive controls, respectively. After incubation at 37 °C for 2 h, the mixtures were centrifuged at 3,000 rpm for 5 min, and the absorbance of the supernatant was measured at 560 nm using a microplate reader. The hemolysis rate was calculated according to the following equation:

$$\begin{aligned}Hemolysis\; rate\;(\%)\\=\:\frac{{\mathrm{O}\mathrm{D}}_{\mathrm{s}\mathrm{a}\mathrm{m}\mathrm{p}\mathrm{l}\mathrm{e}}-{\mathrm{O}\mathrm{D}}_{\mathrm{n}\mathrm{e}\mathrm{g}\mathrm{a}\mathrm{t}\mathrm{i}\mathrm{v}\mathrm{e}\:\mathrm{c}\mathrm{o}\mathrm{n}\mathrm{t}\mathrm{r}\mathrm{o}\mathrm{l}}\:}{{\mathrm{O}\mathrm{D}}_{\mathrm{p}\mathrm{o}\mathrm{s}\mathrm{i}\mathrm{t}\mathrm{i}\mathrm{v}\mathrm{e}\:\mathrm{c}\mathrm{o}\mathrm{n}\mathrm{t}\mathrm{r}\mathrm{o}\mathrm{l}}-{\mathrm{O}\mathrm{D}}_{\mathrm{n}\mathrm{e}\mathrm{g}\mathrm{a}\mathrm{t}\mathrm{i}\mathrm{v}\mathrm{e}\:\mathrm{c}\mathrm{o}\mathrm{n}\mathrm{t}\mathrm{r}\mathrm{o}\mathrm{l}}}\times100\%\end{aligned}$$

During the in vivo experiments, the body weight of each mouse was recorded every other day. On day 12, blood samples and major organs were collected for analysis of routine hematological parameters and key serum biochemical markers. The major organs were weighed, fixed in 4% paraformaldehyde, and subjected to hematoxylin and eosin (H&E) staining to assess potential organ toxicity. Obtained H&E sections were examined on a pathological section scanner (Pannoramic MIDI, 3DHISTECH, Budapest, Hungary). Blood was taken for biochemical analysis and blood routine examination.

## Results and discussion

### Synthesis of the L-BSO prodrug copolymer

We synthesized the L-BSO prodrug copolymer (ELPM) by following the steps provided in Figure S1 [40]. Specifically, to synthesize (S)-4-(prop-2-yn-1-yl) oxazolidine-2,5-dione (PGNCA), the 2-aminopent-4-ynoic acid was reacted with triphosgene in the presence of α-pinene. In the subsequent step, methoxy-terminated oligo (ethylene glycol) amine was conjugated to PGNCA via ring-opening polymerization, yielding a polymer backbone bearing alkyne side chains and covalently linked to the oligo (ethylene glycol) segment. Thereafter, the obtained polymer was reacted with mercaptoethanol under UV irradiation with 2,2-dimethoxy-2-phenylacetophenone (DMPA) as a photoinitiator, thereby introducing hydroxyl-terminated thioether side chains. Finally, the hydroxyl-functionalized polymer was conjugated with L-BSO through an esterification reaction. The carboxyl group of L-BSO was covalently linked to the hydroxyl groups on the polymer side chains, thus successfully incorporating L-BSO into the polymer structure to obtain the final polymer (ELPM). The chemical structures of the products were confirmed by ^1^H NMR (400 MHz) spectroscopy (Figures S2 and S3).

### Preparation and characterization of EsCu@NPs

**Fig. 2:**
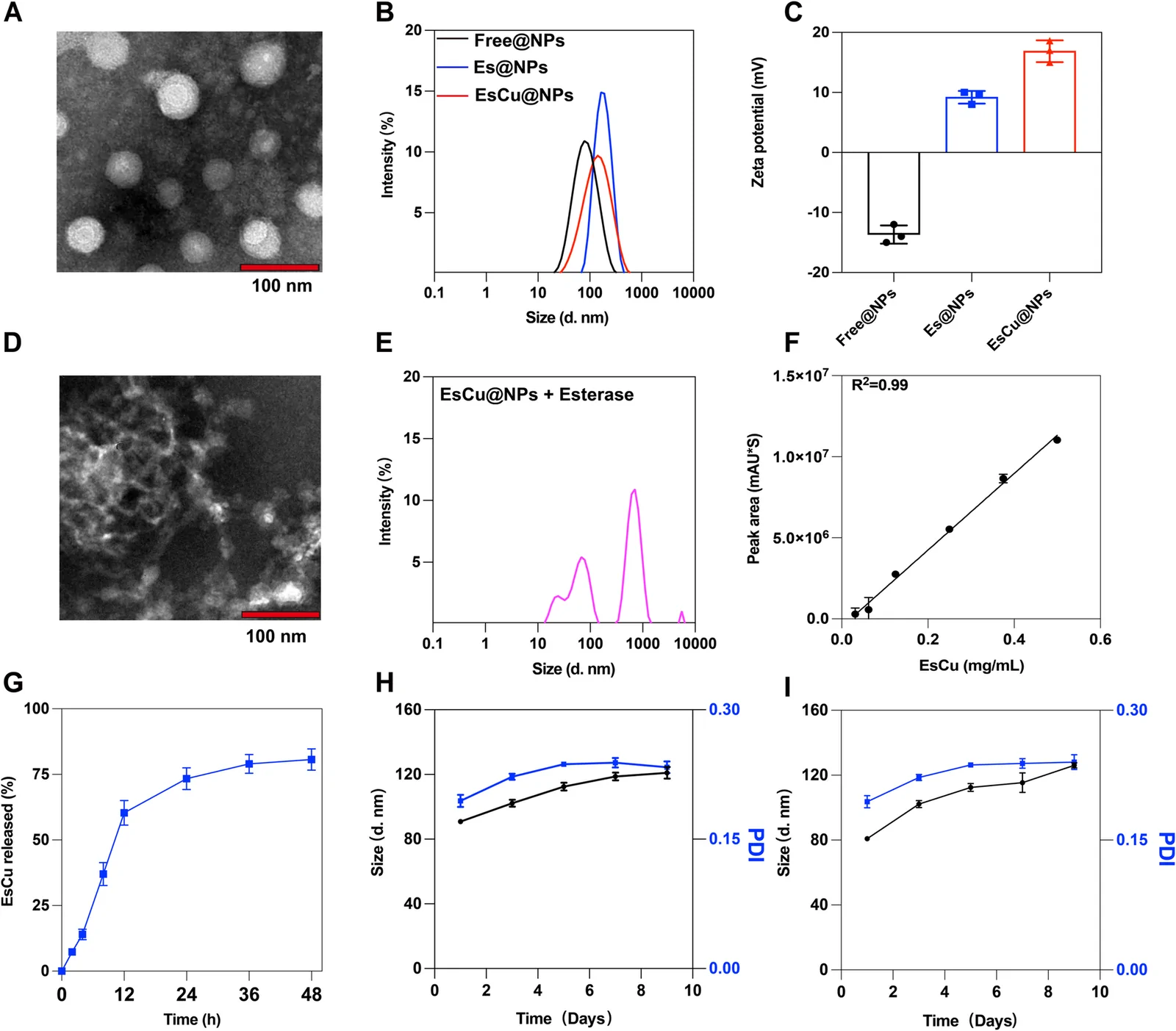
Preparation and characterization of EsCu@NPs. (**A**) TEM image of EsCu@NPs. (**B**) Size profiles of Free@NPs, Es@NPs, and EsCu@NPs. (**C**) Zeta potential profiles of Free@NPs, Es@NPs, and EsCu@NPs. (**D**) TEM image profile of EsCu@NPs after esterase treatment. (**E**) Size profile of EsCu@NPs after esterase treatment. (**F**) The HPLC calibration curve of EsCu (*n* = 3). (**G**) Cumulative release profile of EsCu (*n* = 3). (**H**) Stability of EsCu@NPs in DMEM. (**I**) Stability of EsCu@NPs in PBS. Data are expressed as mean ± SD

In this study, an esterase-sensitive polymeric carrier incorporating L-BSO, termed ELPM, was rationally designed and employed to encapsulate elesclomol–copper complex (EsCu). Through hydrophobic interactions, this system self-assembled into nanoparticles (EsCu@NPs). For comparison, nanoparticles loaded with Es alone (Es@NPs) were fabricated using the same method. The physicochemical properties of both Es@NPs and EsCu@NPs were then systematically investigated. Transmission electron microscopy (TEM) revealed that both formulations exhibited uniform spherical morphology, with average diameters of 55 ± 6 nm (Es@NPs) and 32 ± 5 nm (EsCu@NPs), respectively (Figs. 2A and S4). Dynamic light scattering (DLS) analysis further determined their hydrodynamic diameters to be approximately 197.0 nm (PDI = 0.187) for Es@NPs and 95.0 nm (PDI = 0.206) for EsCu@NPs (Fig. 2B). Zeta potential measurements indicated positive surface charges of + 10.6 mV and + 19.6 mV, respectively (Fig. 2C). These results collectively confirm that both nanoparticles were well dispersed in aqueous solution, and the inclusion of copper facilitated tighter nanoparticle assembly.

The esterase-responsive behavior of EsCu@NPs was attributed to the presence of ester bonds linking L-BSO to the polymer backbone. Upon exposure to esterase, these ester linkages were cleaved, triggering nanoparticle disassembly and the release of encapsulated cargos, including EsCu and L-BSO. TEM imaging further confirmed the structural degradation of EsCu@NPs under esterase treatment (Fig. 2D). This dissociation was next validated by DLS, which revealed a sharp increase in hydrodynamic diameter from 95.0 nm to 255.5 nm in the presence of esterase (Fig. 2E). A substantial increase in Cu release was observed in esterase-containing media (15 U/mL, 24 h) compared with PBS under the same conditions (Figure S5). Similarly, EsCu release from EsCu@NPs was also detected and quantified (Fig. 2F and G), further supporting the esterase-triggered disassembly mechanism. Together, these findings validate the rational design of EsCu@NPs as a smart nanocarrier system for enzyme-responsive drug release.

The colloidal stability of EsCu@NPs in Dulbecco’s Modified Eagle Medium (DMEM) and PBS was next assessed. The particle size remained unchanged for 24 h and exhibited no significant aggregation over a 9-day period (Fig. 2H and I), indicating excellent stability under physiological conditions.

### Cellular uptake and mechanism of enhanced cuproptosis

**Fig. 3:**
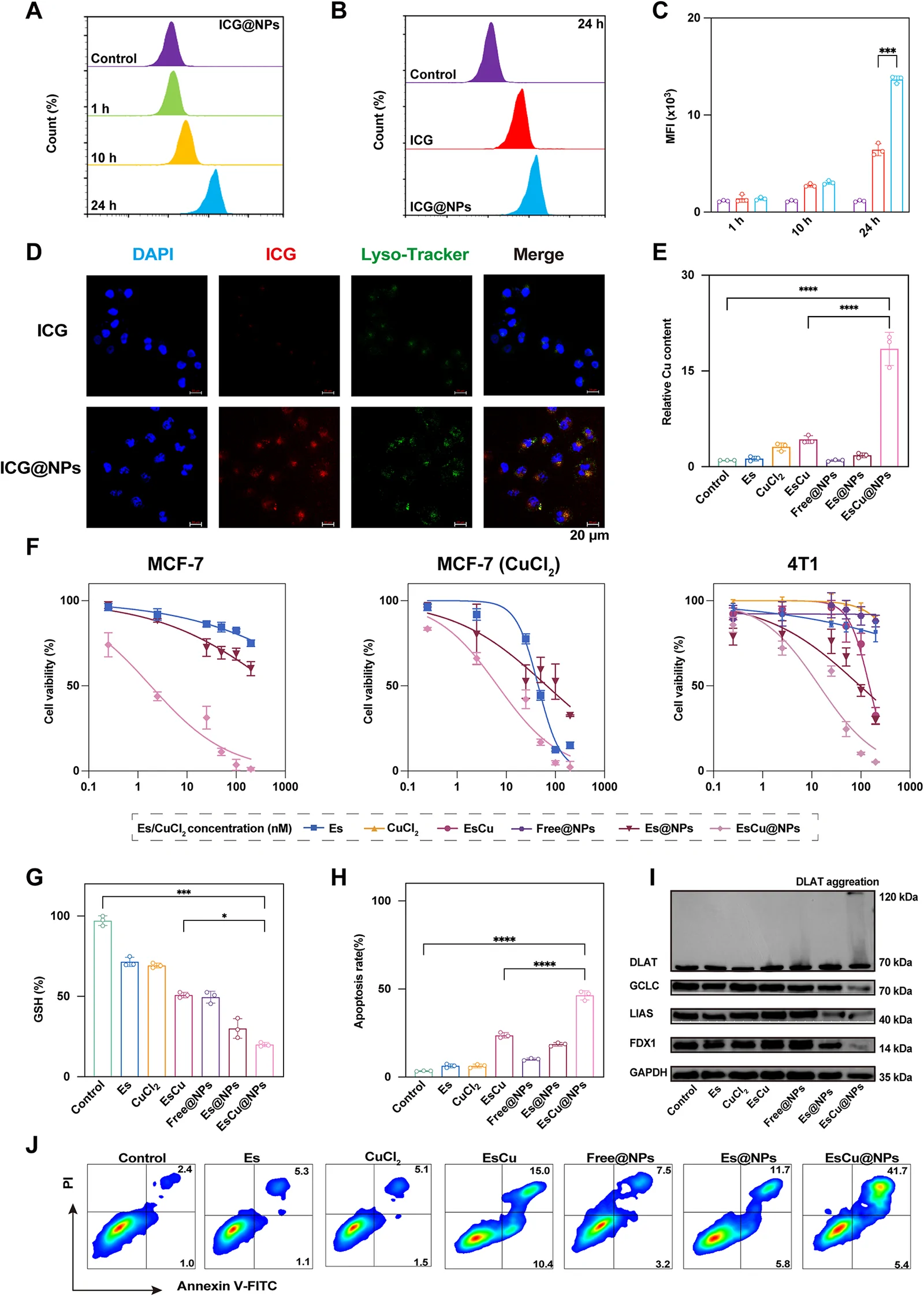
Intracellular drug delivery by EsCu@NPs and in vitro cytotoxicity/cuproptosis mechanism. (**A**) Time-dependent cellular uptake of ICG@NPs in 4T1 cells at 1, 10, and 24 h. (B) Cellular uptake of ICG and ICG@NPs in 4T1 cells at 24 h. (**C**) The quantification of mean fluorescence intensity (MFI). (**D**) CLSM images of 4T1 cells 24 h after exposure to ICG and ICG@NPs. (**E**) Intracellular copper accumulation across treatment groups quantified by ICP (*n* = 3). (**F**) Cell viability of 4T1 and MCF-7 cells with different treatments (*n* = 3). (**G**) Intracellular GSH levels after the indicated treatments (*n* = 3). (**H**) Quantification of apoptosis rates (*n* = 3). (**I**) Western blot analysis of LIAS, FDX1, GCLC, and DLAT after the indicated treatments. (**J**) Apoptosis analysis by Annexin V-FITC/PI staining following different treatments. Data are expressed as mean ± SD. Statistical analysis was performed using one-way analysis of variance (ANOVA) with Tukey test. * *P* ≤ 0.05, ** *P* ≤ 0.01, *** *P* ≤ 0.001, **** *P* ≤ 0.0001

Efficient cellular uptake is essential for nanocarriers to deliver therapeutic payloads, thereby ensuring sufficient intracellular accumulation and facilitating subsequent therapeutic efficacy. To verify cellular internalization, ICG was encapsulated to obtain fluorescent ICG@NPs. Flow cytometry (FCM) analysis demonstrated a clear time-dependent increase in intracellular red fluorescence in 4T1 cells treated with ICG@NPs, with the signal at 24 h markedly higher than at 1 h and 10 h, indicating continuous and efficient uptake (Fig. 3A). Compared with ICG, ICG@NPs exhibited significantly stronger cellular fluorescence at 24 h (Fig. 3B and C). Confocal laser scanning microscopy (CLSM) further corroborated these findings, revealing markedly enhanced intracellular fluorescence with ICG@NPs relative to ICG (Fig. 3D). Having established efficient uptake, we next investigated whether this translated into enhanced intracellular copper delivery.

Because accumulation of copper is a key trigger for cuproptosis, we next quantified intracellular Cu by ICP after treating 4T1 cells with control, Es, CuCl_2_, EsCu, Free@NPs, Es@NPs, and EsCu@NPs. Among these groups, EsCu@NPs produced the highest intracellular Cu levels, demonstrating significantly enhanced Cu delivery (Fig. 3E).

Given that intracellular Cu overload can be counteracted by the abundant GSH pool in tumor cells, we next sought to determine an appropriate L-BSO concentration for GSH depletion. To this end, we evaluated the relationships among L-BSO concentration, intracellular GSH levels, and sensitivity to EsCu treatment in vitro. Intracellular GSH levels decreased in a dose-dependent manner as the L-BSO concentration increased. Notably, 50 µM L-BSO induced substantial GSH depletion, whereas further increasing the concentration to 100 µM resulted in only a limited additional reduction (Figure S6A). In parallel, increasing L-BSO concentrations progressively enhanced the inhibitory effect of EsCu on cell viability (Figure S6B). For EsCu@NPs, an Es-equivalent concentration of 50 nM corresponded to an L-BSO concentration of approximately 47 µM, which closely matched the validated effective L-BSO concentration.

We next examined whether L-BSO-containing nanoparticle formulations effectively depleted intracellular GSH after treatment. Free@NPs, Es@NPs, and EsCu@NPs all markedly reduced cellular GSH levels. Notably, EsCu@NPs induced the most pronounced GSH depletion, with GSH levels significantly lower than those in the other treatment groups **(**Fig. 3G**)**. Together with intracellular Cu accumulation, these biochemical changes suggest that EsCu@NPs simultaneously promote Cu delivery and weaken GSH-mediated Cu buffering, thereby creating favorable conditions for enhanced copper-dependent cytotoxicity. We therefore further examined whether canonical hallmarks of cuproptosis were activated.

MTT assays demonstrated the superior cytotoxicity of EsCu@NPs compared with CuCl_2_ or Es alone (*P* ≤ 0.001, Fig. 3F). Without CuCl_2_ pretreatment, the IC_50_ values of Es and Es@NPs in MCF-7 and 4T1 cells exceeded 200 nM, indicating limited activity. Pretreating MCF-7 cells with 0.1 µM CuCl_2_ significantly reduced the IC_50_ values of Es and Es@NPs. In 4T1 cells, EsCu@NPs achieved the lowest IC_50_ value (14.22 nM), markedly lower than EsCu or Es@NPs, consistent with efficient Cu delivery and enhanced cytotoxicity. Annexin V-FITC/PI staining was further performed to evaluate apoptosis-associated membrane alterations and the overall cell death profile after treatment. EsCu@NPs group showed a markedly increased the apoptotic rate compared with EsCu or Es@NPs groups **(**Fig. 3H and J**)**.

To investigate whether Cu accumulation induced by EsCu@NPs could trigger intracellular toxic protein aggregation, we next examined mitochondrial events. Cu^2+^ released from the nanomaterial was reduced to Cu⁺ by FDX1 and subsequently transferred to lipoylated DLAT, inducing DLAT oligomerization. The resulting increase in insoluble DLAT led to proteotoxic stress and ultimately cell death. Consistently, Cu⁺ binding to DLAT was observed, accompanied by toxic protein aggregation within cells. Western blot analysis further confirmed that the expression levels of LIAS, FDX1, and GCLC were lowest in the EsCu@NPs group and pronounced DLAT aggregation was evident (Fig. 3I). Notably, suppression of GCLC is consistent with the action of L-BSO, a specific inhibitor of glutamate–cysteine ligase, thereby blocking GSH biosynthesis. Collectively, these findings demonstrate that EsCu@NPs enhance cellular uptake, increase intracellular Cu while depleting GSH, and activate the biochemical hallmarks of cuproptosis, culminating in potent Cu-dependent antitumor activity in vitro.

Given that GSH depletion may activate compensatory antioxidant responses and thereby weaken cuproptosis induction, we next examined whether the thioredoxin (TXN) antioxidant pathway was activated after EsCu@NPs treatment [41]. Thioredoxin reductase 1 (TXNRD1), a key enzyme of the TXN antioxidant system, was not significantly upregulated after EsCu@NPs treatment (Figure S7). Meanwhile, intracellular ROS levels remained markedly elevated, particularly in the EsCu@NPs group (Figure S8). Together, these results suggest that antioxidant compensation through the TXNRD1-related TXN pathway was insufficient to weaken EsCu@NPs-induced cuproptosis.

### EsCu@NPs reprogram the TIME

**Fig. 4:**
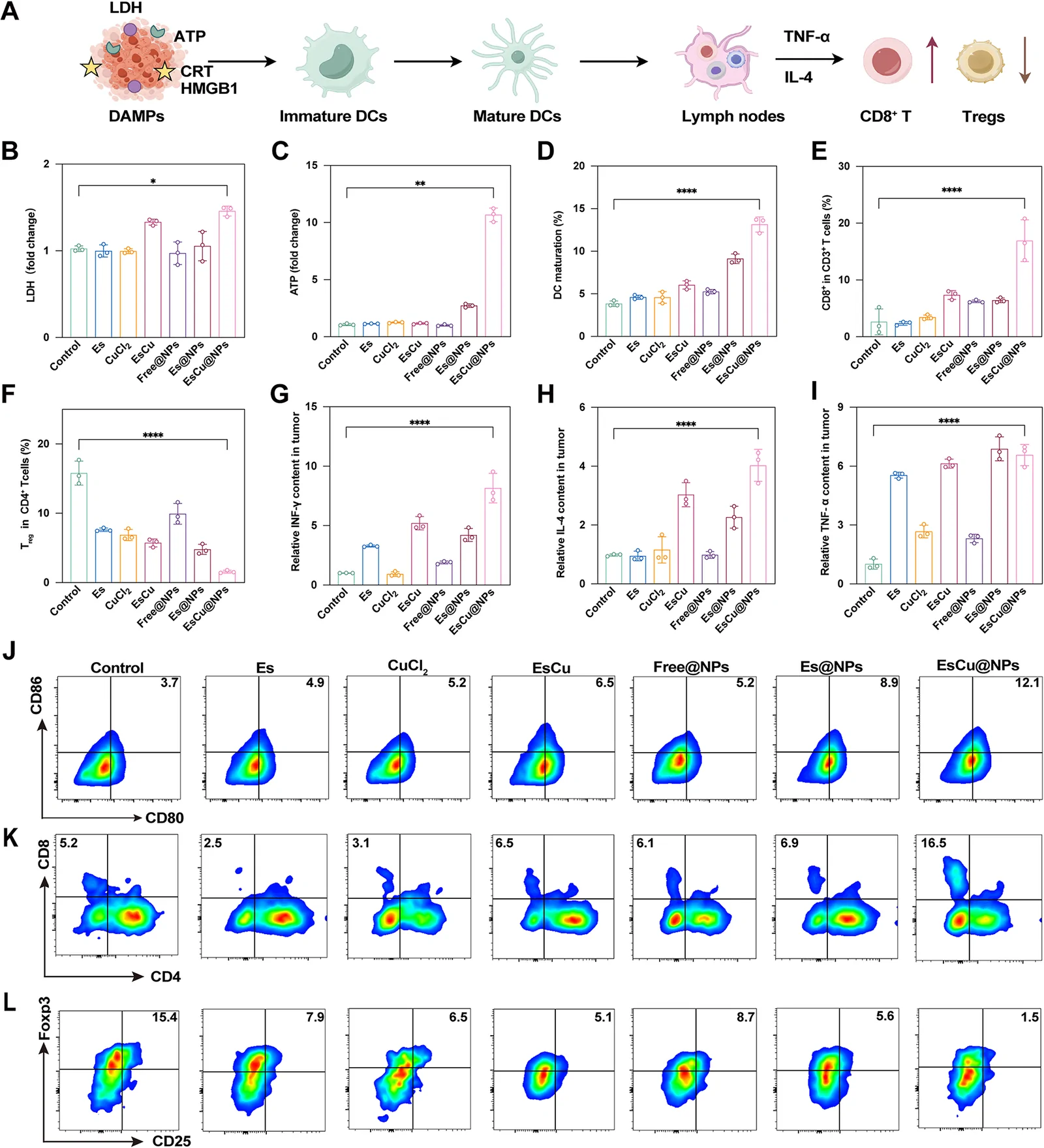
EsCu@NPs induce ICD and reprogram the immunosuppressive TIME. (**A**) Schematic illustration of EsCu@NPs inducing ICD and remodeling the immunosuppressive TIME. (**B**) Levels of LDH release after different treatments in 4T1 cells (*n* = 3). (**C**) Levels of ATP release after different treatments in 4T1 cells (*n* = 3). (**D**) Quantitative analysis of matured DCs, (**E**) the proportion of infiltrating CD8⁺ T cells among CD3⁺ T cells, (**F**) and the proportion of Tregs among CD4⁺ T cells in the 4T1 TIME following different treatments (*n* = 3 mice). Secretion of IFN-γ (**G**), IL-4 (**H**), and TNF-α (**I**) in the 4T1 TIME after different treatments (*n* = 3 mice). Representative FCM dot plots of mature DCs (**J**), infiltrating CD8⁺ T cells (**K**), and Tregs (**L**). Data are expressed as mean ± SD. Statistical analysis was performed using one-way analysis of variance (ANOVA) with Tukey test. * *P* ≤ 0.05, ** *P* ≤ 0.01, *** *P* ≤ 0.001, **** *P* ≤ 0.0001

Having established that EsCu@NPs potently induce cuproptosis and consequent cytotoxicity, we next interrogated the immunogenicity of this death program and its capacity to remodel the TIME. Accordingly, we assayed canonical hallmarks of ICD, including calreticulin (CRT) membrane translocation, extracellular high mobility group box 1 (HMGB1) and ATP release, LDH leakage, and profiled intratumoral immune subsets to delineate downstream TIME reprogramming (Fig. 4A).

As shown in Figure S9, only weak CRT-positive fluorescence was detected in the control, Es, and CuCl_2_ groups. In contrast, EsCu and EsCu@NPs treatments increased membrane-exposed CRT fluorescence. Meanwhile, intracellular HMGB1 signal was clearly reduced in the EsCu@NPs group, indicating increased HMGB1 release from 4T1 cells. Immunostaining further showed that EsCu@NPs markedly induced CRT membrane translocation (Figure S10), while intracellular HMGB1 staining was significantly diminished (Figure S11), further supporting the activation of ICD. Extracellular ATP and LDH release were quantified using commercial assay kits. Relative to other groups, EsCu@NPs group significantly increased LDH release and extracellular ATP (Fig. 4B and C). These results substantiate that EsCu@NPs robustly induce ICD, thereby bridging tumor cell death and downstream immune activation. Consistent with the induction of ICD, EsCu@NPs also markedly promoted BMDC maturation in vitro, as evidenced by the highest proportion of mature BMDCs among all groups (Figure S12). These results further indicated that EsCu@NPs-induced tumor cell death is immunostimulatory and capable of triggering downstream immune activation.

We further profiled the TIME using a 4T1 subcutaneous syngeneic model. Mice received the indicated treatments, tumors were harvested 9 days post-dosing, and flow cytometry was performed. Compared with control group (Fig. 4D and J), EsCu@NPs markedly enhanced the proportion of mDCs. This result indicates that the EsCu@NPs not only protect and co-deliver active components but also promote copper-mediated immunostimulation, thereby facilitating antigen presentation and T-cell priming. Consistently, EsCu@NPs led to a pronounced increase in intratumoral CD8⁺ T-cell infiltration (Fig. 4E and K), accompanied by a notable reduction in Tregs (Fig. 4F and L). Meanwhile, a marked infiltration of CD8⁺ T cells was also observed within the tumor tissues (Figure S13). The concurrent elevation of effector CD8⁺ T cells with depletion of Tregs suggests a more favorable effector/suppressor balance within the TIME.

To evaluate whether EsCu@NPs-mediated GSH modulation induced systemic immune dysfunction, we analyzed splenic CD8⁺ T cells homeostasis and exhaustion after treatment [42, 43]. EsCu@NPs did not markedly reduce intracellular GSH levels in peripheral CD8⁺ T cells isolated from the spleen and lymph nodes (Figure S14). In addition, EsCu@NPs did not reduce the proportion of CD8⁺ T cells among splenic CD3⁺ T cells, and the proportions of PD-1⁺TIM-3⁺ and PD-1⁺LAG-3⁺ splenic CD8⁺ T cells were not significantly increased. These results suggested that EsCu@NPs did not induce detectable peripheral CD8⁺ T cells reduction or exhaustion (Figure S15).

Given that PD-1 is frequently upregulated on tumor-infiltrating CD8⁺ T cells within the suppressive TIME and its co-expression with TIM-3 or LAG-3 is associated with T-cell exhaustion, we further examined whether EsCu@NPs induced exhaustion-related phenotypes in tumor-infiltrating CD8⁺ T cells. EsCu@NPs markedly reduced the proportions of PD-1⁺CD8⁺ T cells, as well as PD-1⁺TIM-3⁺ and PD-1⁺LAG-3⁺ CD8⁺ T cells subsets in tumor tissues (Figure S16). The gating strategy and flow cytometry workflow were illustrated in Figures S17 and S18.

To determine whether this reflected functional activation rather than mere infiltration, cytokine levels were assessed in tumor lysates. EsCu@NPs substantially increased IFN-γ, IL-4, and TNF-α compared with all other groups (Fig. 4G and I). Both copper and the nanocarrier contributed to this effect, with EsCu outperforming Es and EsCu@NPs further surpassing Es@NPs. These changes highlight a remodeled cytokine milieu supportive of enhanced antigen presentation and cytotoxic T-cell activity. In summary, EsCu@NPs convert cuproptosis into ICD, accompanied by dendritic cell maturation, enhanced CD8⁺ T cells infiltration and depletion of Tregs. Collectively, these effects reprogram the immunosuppressive TIME and provide a strong rationale for neoadjuvant combination with aPD-1 therapy.

### In vivo antitumor efficacy of EsCu@NPs

**Fig. 5:**
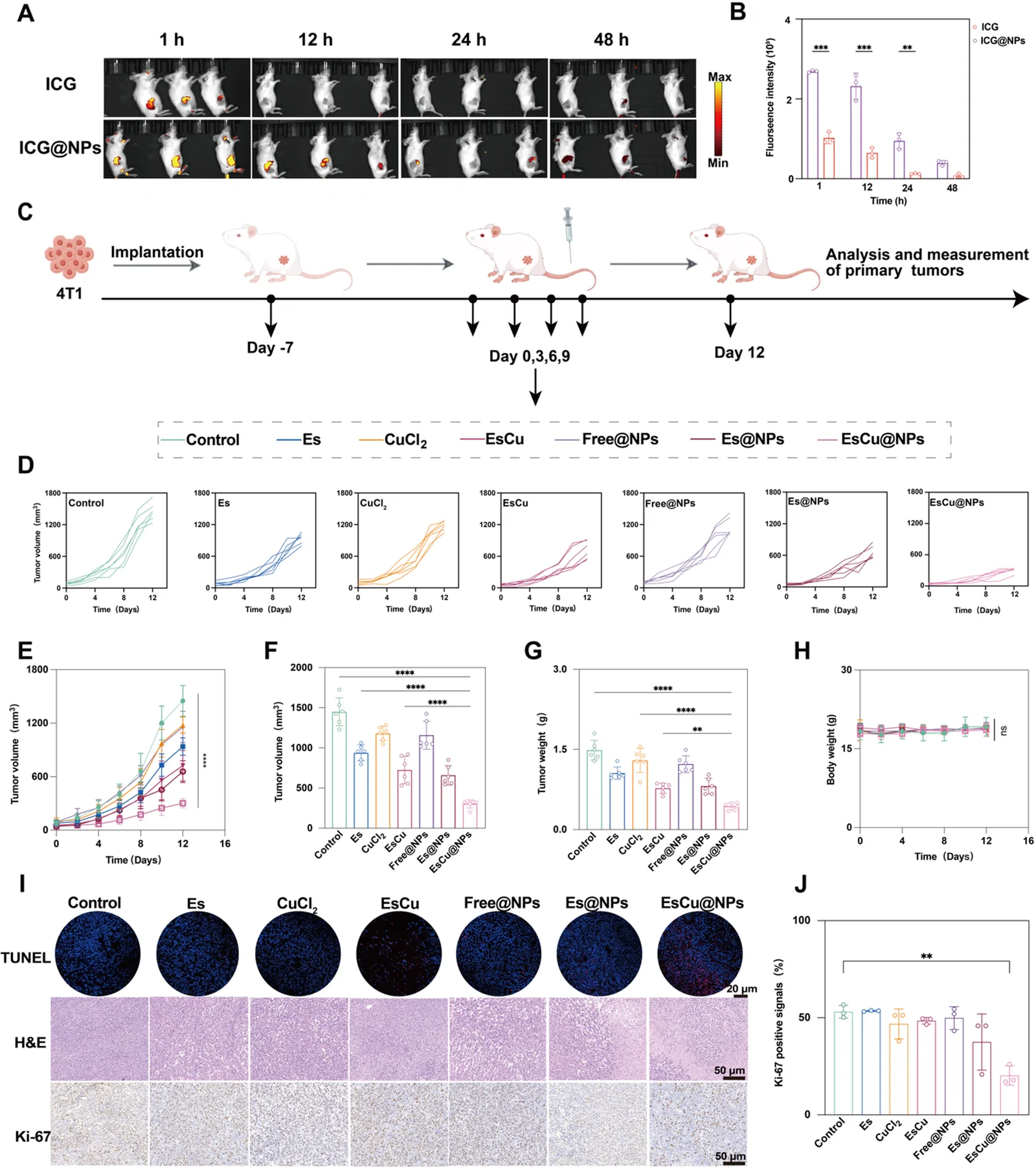
In vivo distribution of ICG@NPs and antitumor efficacy of EsCu@NPs in the 4T1 model. Whole-body IVIS fluorescence imaging (**A**) and quantitative analysis (**B**) show the in-vivo distribution of ICG and ICG@NPs (*n* = 3 mice). (**C**) Schematic of the dosing and efficacy-evaluation workflow for EsCu@NPs in the 4T1 syngeneic tumor model. (**D-F**) Tumor growth curves under the indicated treatments (*n* = 6 mice). (**G**) Terminal tumor weight at study end (*n* = 6 mice). (**H**) Body-weight changes during treatment (*n* = 6 mice). (**I**) Representative tumor histology: TUNEL, H&E, and Ki-67 staining (scale bars as indicated). (**J**) Percentage of Ki-67 positive signals following different treatments (*n* = 3). Data are expressed as mean ± SD. Statistical analysis was performed using one-way analysis of variance (ANOVA) with Tukey test. ns *P*>0.05, * *P* ≤ 0.05, ** *P* ≤ 0.01, *** *P* ≤ 0.001, **** *P* ≤ 0.0001

Building on evidence of cuproptosis-driven ICD and TIME remodeling, we next determined whether EsCu@NPs accumulate in tumors in vivo and confer tangible therapeutic benefit, beginning with IVIS tracking followed by efficacy readouts. We first used IVIS to assess tumor uptake and retention of ICG and ICG@NPs. As shown in Fig. 5A and B, the tumor fluorescence of ICG declined rapidly at 1 h post-injection, whereas ICG@NPs remained essentially stable over the first 12 h. Ex vivo imaging at 48 h revealed pronounced accumulation of ICG@NPs in tumors (Figure S19), indicating effective tumor-targeted delivery by the nanocarrier.

To evaluate the pharmacokinetic profile and in vivo delivery efficiency of EsCu@NPs, we first quantified copper content in tumor tissues after different treatments. As shown in Figure S20A, the EsCu@NPs group exhibited the highest copper accumulation in tumors, reaching 8.7% ID/g, which was significantly higher than that in the EsCu group (4.9% ID/g) and the Free@NPs group (2.9% ID/g). We next investigated the pharmacokinetic profiles of EsCu and EsCu@NPs in vivo. EsCu displayed relatively high blood levels at early time points (1–5 min), but declined rapidly thereafter, indicating fast systemic clearance (Figure S20B). In contrast, EsCu@NPs markedly slowed the initial decline and maintained higher blood levels from 30 min onward, with significant differences observed at 30, 60, 360, and 720 min compared with EsCu. Notably, EsCu@NPs remained detectable at 24 h, whereas EsCu was nearly eliminated. Collectively, these results demonstrate that EsCu@NPs improved tumor copper delivery efficiency, thereby providing EsCu with a more sustained systemic exposure profile.

In the 4T1 subcutaneous syngeneic model, treatment efficacy was assessed by tumor growth outcomes (Fig. 5C). On day 12, only minimal effects were observed in the Es, CuCl_2_, and Free@NPs groups compared with the control group (Fig. 5D and F). The EsCu and Es@NPs groups showed moderate inhibition, whereas the EsCu@NPs group exhibited the most pronounced suppression of tumor growth, markedly outperforming both Free@NPs (*P* < 0.0001) and Es@NPs (*P* = 0.0009). Tumor-weight measurements confirmed these findings (Fig. 5G), with the EsCu@NPs group achieving the strongest inhibitory effect among all treatment groups. Meanwhile, ICP analysis was performed to determine the copper ion content in tumor tissues from different treatment groups. As shown in Figure S21, tumors from the EsCu@NPs group exhibited significantly higher copper levels compared with other groups. No significant body-weight loss was observed across groups (Fig. 5H), indicating acceptable tolerability. In addition, biochemistry assays (Figure S22), H&E staining (Figure S23) and organ index analyses (Figure S24) performed after the treatment showed that all parameters were within normal ranges, demonstrating that EsCu@NPs exhibited good biocompatibility in vivo without noticeable adverse effects on physiological functions. In the hemolysis assays, the hemolysis rate of EsCu@NPs was less than 5% even at a high concentration of 2 mg/mL (Figure S25).

Western blot analysis of tumor tissue showed that EsCu@NPs markedly promoted the formation of high-molecular-weight DLAT aggregates, accompanied by decreased FDX1 and LIAS expression (Figure S26). IHC staining showed that EsCu@NPs markedly increased DLAT-positive signals, while FDX1 and LIAS signals were reduced after treatment (Figure S27). These results suggest that EsCu@NPs activated cuproptosis-associated mitochondrial protein stress in vivo. Histology provided mechanistic corroboration (Figs. 5I and S28), EsCu@NPs showed the strongest TUNEL positivity together with the lowest Ki-67 index (Fig. 5J). Collectively, efficient tumor delivery coupled with amplified cuproptosis-to-ICD signaling enabled deep tumor debulking and proliferation control in vivo with favorable tolerability, highlighting the therapeutic promise of EsCu@NPs (Figure S29).

### EsCu@NPs as a neoadjuvant to suppress tumors and metastasis

**Fig. 6:**
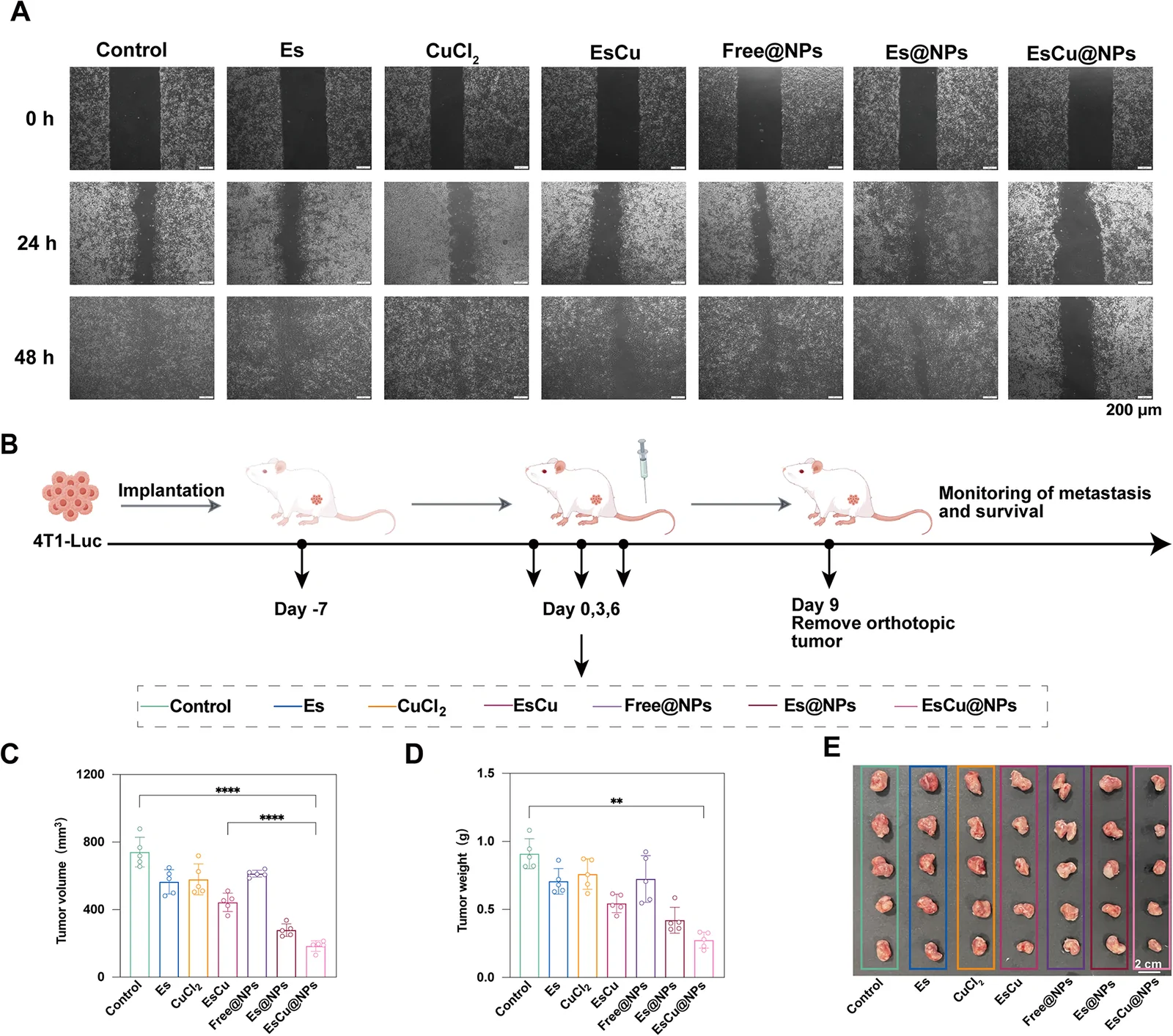
Neoadjuvant EsCu@NPs suppresses orthotopic TNBC. (**A**) Wound-healing assay of 4T1 cells under the indicated treatments; migration quantified as wound-closure rate at 0, 24, and 48 h. (**B**) Anti-tumor experimental procedure of BALB/c mice. The average volumes (**C**) and weights (**D**) of the orthotopic tumors after the treatments (*n* = 5 mice). (**E**) Representative photographs of excised tumors from each group. Data are expressed as mean ± SD. Statistical analysis was performed using one-way analysis of variance (ANOVA) with Tukey test. ns *P* > 0.05, * *P* ≤ 0.05, ** *P* ≤ 0.01, *** *P* ≤ 0.001, **** *P* ≤ 0.0001

Given the aggressiveness of TNBC and its high propensity for postoperative recurrence and distant metastasis, effective strategies to control both primary tumors and metastatic dissemination are urgently needed. This combined cytotoxic and immunomodulatory activity not only directly suppresses primary tumor growth but also generates systemic antitumor immunity capable of targeting disseminated tumor cells, providing a mechanistic rationale for EsCu@NPs to prevent postoperative recurrence and distant metastasis. Additionally, as shown in Fig. 6A and S30, cell scratch assay revealed that EsCu@NPs reduced 4T1 wound-closure rates from 58% (control) to 13% (EsCu@NPs) at 24 h, and from 100% (control) to 25% (EsCu@NPs) at 48 h, indicating significant suppression of cell migration. Building on these findings, we implemented EsCu@NPs as a neoadjuvant regimen prior to resection: preoperative dosing downstaged and immunologically pre-heated tumors, reduced minimal residual disease, curtailed postsurgical recurrence, spontaneous lung metastasis, and ultimately prolonged survival in the orthotopic 4T1 model.

To further evaluate the ability of EsCu@NPs to inhibit distant metastasis, an orthotopic TNBC model was established by injecting 4T1-Luc cells into the mammary fat pads of female BALB/c mice. When the tumor volume in each mouse reached approximately 100 mm^3^, the mice were randomly divided into seven groups and treated with control, Es, EsCu, CuCl_2_, Free@NPs, Es@NPs and EsCu@NPs on days 0, 3, and 6 via intravenous injection. The orthotopic 4T1-Luc tumors were surgically removed and weighed on day 9, while the development of spontaneous lung metastases was monitored with an IVIS spectrum **(**Fig. 6B**)**. Following three rounds of systemic treatment, EsCu@NPs group exhibited robust and sustained antitumor activity in the orthotopic TNBC model. In terms of tumor growth inhibition, EsCu@NPs group achieved the most remarkable effect, with an average tumor volume of only 184 ± 31 mm^3^ (Fig. 6C), corresponding to 24.8% of that in the control group (740 ± 87 mm^3^). Similarly, the mean tumor weight in the EsCu@NPs group were reduced to 0.27 ± 0.06 g (Fig. 6D), which was only 29.7% of the control group (0.91 ± 0.11 g). However, the tumors grew quickly in the control, Es, EsCu, CuCl_2_, Free@NPs, Es@NPs groups, associating with large tumor volumes and higher tumor weights (Fig. 6E). These results quantitatively confirm the pronounced reduction in tumor burden and highlight the potent capacity of EsCu@NPs to suppress primary tumor progression.

**Fig. 7:**
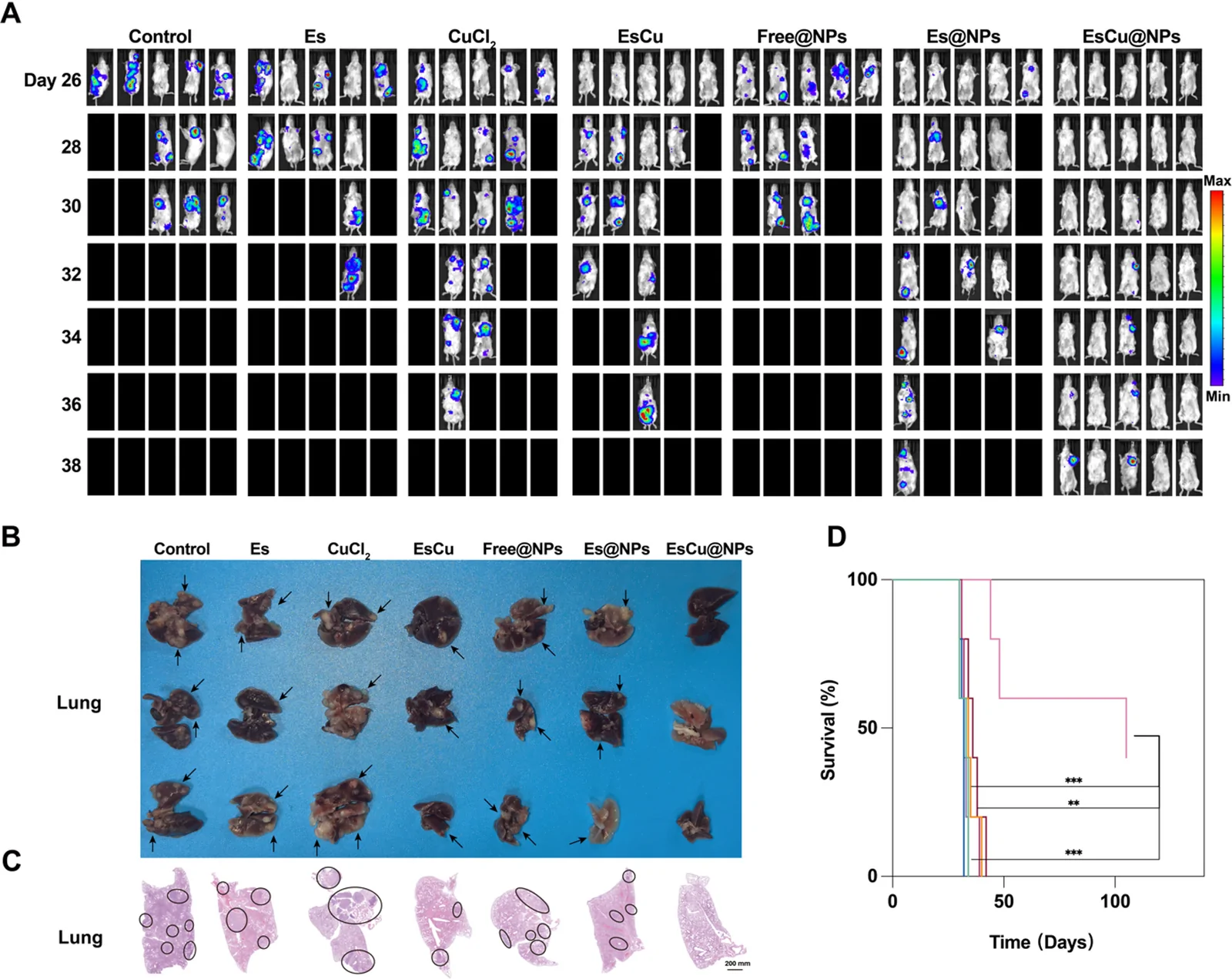
Neoadjuvant EsCu@NPs inhibit metastasis and recurrence in orthotopic TNBC. (**A**) IVIS images show spontaneous lung metastases from the orthotopic 4T1-Luc tumors in each group. Black panels indicate mice that had died or were euthanized due to tumor burden. (**B**) Ex vivo images show lungs with metastatic breast tumors (*n* = 3 mice). Black arrows and circles point to lung metastatic foci. (**C**) H&E staining shows lung metastasis in mice. (**D**) Survival rates of mice after receiving the different treatments (*n* = 5 mice, two-tailed Mantel-Cox test). Data are expressed as mean ± SD. ns *P* > 0.05, * *P* ≤ 0.05, ** *P* ≤ 0.01, *** *P* ≤ 0.001, **** *P* ≤ 0.0001

Importantly, EsCu@NPs group also provided durable control of postsurgical disease. In the control group, rampant systemic dissemination appeared on day 28, leading to precipitous mortality; most of the mice in the control, Es, EsCu, CuCl_2_, Free@NPs, and Es@NPs groups succumbed before day 34, with deaths confirmed to involve lung metastasis (Fig. 7A and B). Metastatic progression was modestly delayed in the Es and CuCl_2_ groups but not prevented. Intermediate postsurgical control was observed in the EsCu and Es@NPs groups, with new lesions emerging at approximately day 30; by day 40, all mice had experienced recurrence or metastasis and subsequently died **(**Fig. 7C**)**. Strikingly, the EsCu@NPs group achieved 60% survival at day 100 (Fig. 7D), indicating meaningful extension of survival period. Overall, the EsCu@NPs-based neoadjuvant therapy to effectively eradicate the primary TNBC and suppress its lung metastasis.

### EsCu@NPs combined with aPD-1 synergistically enhance antitumor efficacy

Building upon the capacity of EsCu@NPs to remodel the immunosuppressive TIME through ICD induction, DC maturation, CD8⁺ T-cell recruitment, and Tregs depletion, we next explored their potential to synergize with immune checkpoint blockade. PD-1 plays a central role in tumor immune evasion by suppressing T-cell activity within the TIME. Although PD-1 blockade has revolutionized cancer therapy, its efficacy in cold tumors such as TNBC remains limited due to insufficient T-cell priming and immunosuppressive TIME constraints. By inducing ICD and enhancing antigen presentation, EsCu@NPs provide an ideal strategy to convert cold tumors into hot tumors, thereby creating a favorable immunological context for PD-1 blockade. TNBC poses substantial challenges to immunotherapy due to its inherently low immunogenicity and limited responsiveness to immune-based treatments. EsCu@NPs have demonstrated a remarkable capacity to remodel the immunosuppressive TIME of TNBC, thereby enhancing T-cell infiltration and antigen presentation. Based on these effects, the combination of EsCu@NPs with PD-1 blockade holds strong potential to significantly improve immunotherapeutic efficacy in TNBC by expanding the responsive patient population, enhancing therapeutic outcomes, and delaying resistance.

**Fig. 8:**
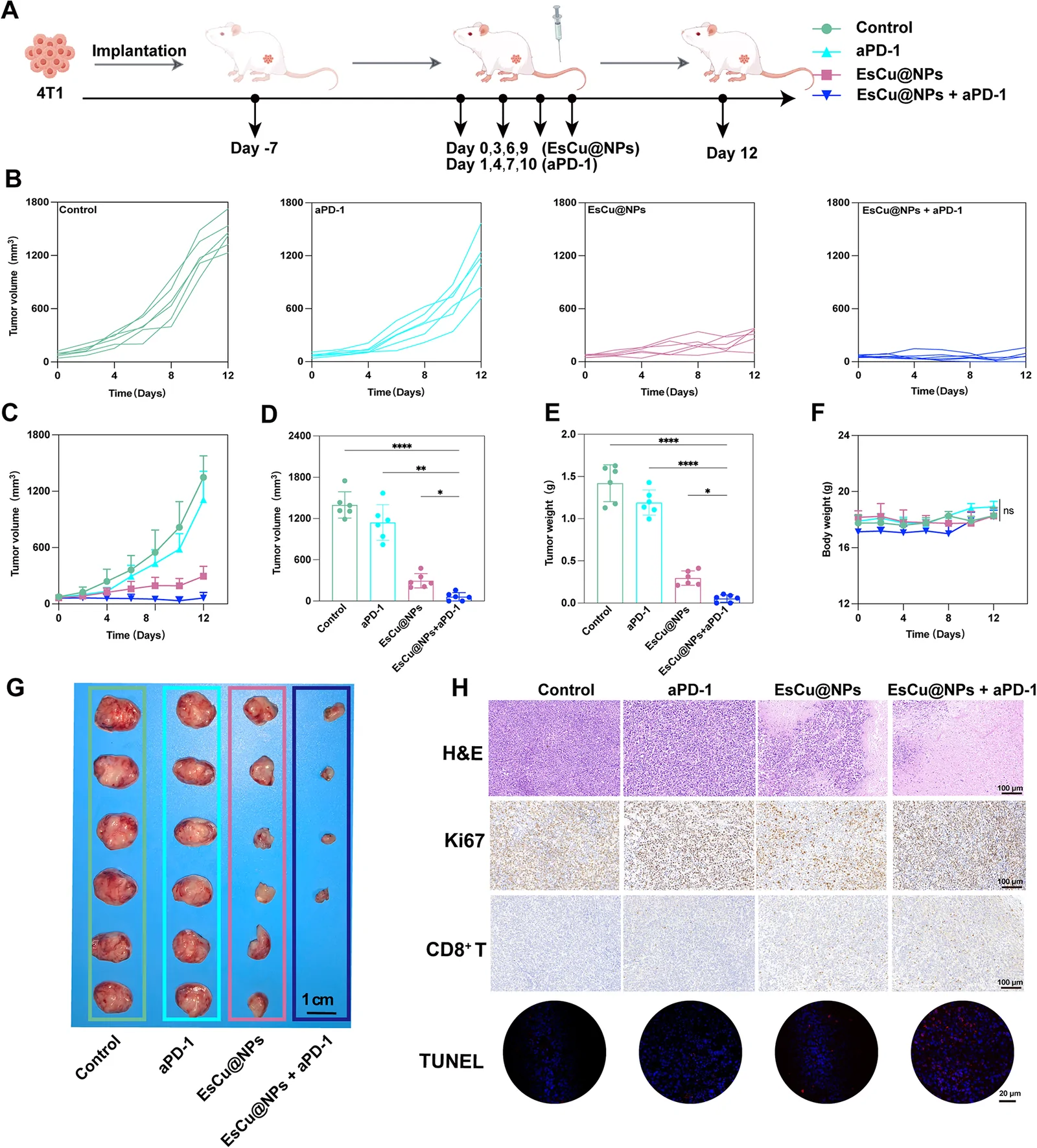
Therapeutic efficacy of EsCu@NPs combined with aPD-1. (**A**) Schematic illustration of EsCu@NPs combined with aPD-1 treatment. (**B-D**) Tumor growth curves of mice in different treatment groups (*n* = 6 mice). (**E**) Tumor weights at the end of treatment (*n* = 6 mice). (**F**) Body weight changes of mice during treatment (*n* = 6 mice). (**G**) Representative images of excised tumors after treatment (*n* = 6 mice). (**H**) Histological analysis of tumor tissues by H&E staining, Ki-67 immunostaining, CD8⁺ T-cell infiltration, and TUNEL staining. Data are expressed as mean ± SD. Statistical analysis was performed using one-way analysis of variance (ANOVA) with Tukey test. ns *P*>0.05, * *P* ≤ 0.05, ** *P* ≤ 0.01, *** *P* ≤ 0.001, **** *P* ≤ 0.0001

Subsequently, a 4T1 subcutaneous tumor model was established to evaluate the antitumor effect of EsCu@NPs in combination with aPD-1. Mice were intravenously administered control, aPD-1, EsCu@NPs (Es, 6 mg/kg), and EsCu@NPs + aPD-1 four times, respectively (Es, 6 mg/kg; aPD-1, 10 mg/kg) (Fig. 8A). As shown in Fig. 8B and D, tumor growth was rapid in the control and aPD-1 groups, whereas the EsCu@NPs group exhibited moderately slower tumor progression, indicating a modest antitumor effect. Notably, the combination of EsCu@NPs and aPD-1 markedly suppressed tumor growth. At the end of treatment, the superior antitumor efficacy of the EsCu@NPs + aPD-1 group was further confirmed by tumor excision and weighing (Fig. 8E), accompanied by representative tumor images (Fig. 8G). During the treatment period, mice maintained stable body weight (Fig. 8F), indicating that the treatment regimen generally preserved their physiological condition. In addition, blood biochemical analyses (Figure S31), H&E staining (Figure S32) and organ index (Figure S33) analyses performed after the treatment showed that all parameters were within normal ranges, demonstrating that EsCu@NPs + aPD-1 exhibited good biocompatibility in vivo without noticeable adverse effects on physiological functions. To further assess therapeutic efficacy, tumor sections were analyzed using H&E staining and TUNEL. The EsCu@NPs + aPD-1 group exhibited pronounced nuclear contraction and chromatin condensation (Figs. 8H, S34 to S36), confirming its potent tumor cell-killing effect. Meanwhile, tumors treated with EsCu@NPs + aPD-1 showed significantly higher infiltration of CD8⁺ T cells compared with other groups. These in vivo results were consistent with the in vitro findings, further supporting the feasibility of EsCu@NPs + aPD-1 for cancer therapy. In summary, EsCu@NPs + aPD-1 effectively reprogrammed the TIME and elicited a robust antitumor immune response, validating the therapeutic potential of this combination strategy for TNBC treatment.

## Conclusion

The TIME of TNBC represents a complex ecosystem composed of various immune cells, signaling molecules, and tumor cells, whose dynamic balance profoundly affects tumor progression, metastasis, and therapeutic responsiveness [44]. TNBC is characterized by high expression of immune-related genes, yet concurrently overexpresses multiple immunosuppressive markers, indicating the coexistence of strong immune activation and suppression [45, 46]. The balance between these forces determines the ultimate immune phenotype. Although ICB has shown promising efficacy in theory [47], its clinical benefits in TNBC remain limited, as demonstrated by the poor response to aPD-1 monotherapy in 4T1 tumor models [48]. The immunosuppressive TIME is thus a major obstacle to the success of ICB. Combining ICB with tumor microenvironment-modulating strategies provides a rational avenue to overcome this limitation [49].

Cuproptosis represents a newly recognized form of programmed cell death triggered by copper accumulation and subsequent proteotoxic stress, leading to metabolic collapse [50]. Emerging evidence indicates that the expression of cuproptosis-related genes correlates strongly with immune characteristics in TNBC. For instance, elevated FDX1 expression is associated with increased CD8⁺ T-cell infiltration, reduced immunosuppressive molecule expression, and improved patient survival, suggesting that FDX1-dependent cuproptosis may promote antitumor immunity [51, 52]. These findings support the potential of targeting cuproptosis to actively modulate the immune state of the TIME. Unlike conventional cytotoxic or redox-based metal therapies, which often suffer from poor tumor selectivity and systemic toxicity [53], cuproptosis-based strategies provide a metabolically specific and immunologically favorable mechanism of tumor eradication [54].

Guided by these insights, we engineered EsCu@NPs, an esterase-responsive nanoplatform that integrates copper-induced metabolic stress with immunotherapy sensitization. By covalently incorporating an L-BSO prodrug to suppress glutathione synthesis and encapsulating an elesclomol–copper complex to ensure efficient copper delivery, EsCu@NPs overcome redox resistance and enable tumor-selective cuproptosis. Importantly, this metabolic perturbation is converted into ICD, leading to remodeling of the immunosuppressive tumor microenvironment and enhanced responsiveness to PD-1 blockade. Collectively, EsCu@NPs enable a reprogram-first, then-strike approach that links cuproptosis induction with immunotherapy in TNBC in a mechanistically grounded and translationally relevant manner.

## Supplementary Information

Supplementary Material 1

## Data Availability

No datasets were generated or analysed during the current study.

## Abbreviations

DLAT: Dihydrolipoamide S-acetyltransferase
FDX1: Ferredoxin 1
GCLC: Glutamate-cysteine ligase catalytic subunit
ICB: Immune checkpoint blockade
ICD: Immunogenic cell death
L-BSO: L-Buthionine-(S,R)-sulfoximine
LIAS: Lipoic acid synthetase
SLC31A1: Solute carrier family 31
TIME: Tumor immune microenvironment
TNBC: Triple-negative breast cancer
