# Supplementary material for: Glutathione-depleting self-immolative nanoparticles boost cuproptosis-driven metalloimmunotherapy for triple-negative breast cancer
Source: J Nanobiotechnology. 2026 Jul 7;24:912. doi: 10.1186/s12951-026-04668-9 (PMC13625500; doi:10.1186/s12951-026-04668-9)
Supplement: Supplementary file 1 [file 12951_2026_4668_MOESM1_ESM.docx]

Supporting Information for

**Glutathione-Depleting Self-Immolative Nanoparticles Boost Cuproptosis-Driven Metalloimmunotherapy for Triple-Negative Breast Cancer**

Yunhai Fu^1^, Xiaoyu Wang^1^, Yinqi Li^1^, Xiaodong Yang^1^, Ziying Yao^1^, Jingwen Wang^1^, Yaxin Zhang^1^, Tianping Li^1 2^, Yunke Zhu^3 4^*, Zhiyao He^1 2^*

^1^Department of Pharmacy, Cancer Center and State Key Laboratory of Biotherapy, West China Hospital, Sichuan University, Chengdu 610041, China.

^2^Key Laboratory of Drug-Targeting and Drug Delivery System of the Education Ministry and Sichuan Province, Sichuan Engineering Laboratory for Plant-Sourced Drug and Sichuan Research Center for Drug Precision Industrial Technology, West China School of Pharmacy, Sichuan University, Chengdu 610041, China.

^3^Institute of Thoracic Oncology and Department of Thoracic Surgery, West China Hospital, Sichuan University, Chengdu 610041, China.

^4^Department of Thoracic Surgery, Chengdu Shangjin Nanfu Hospital, Chengdu 611730, China.

***Corresponding Authors:** Yunke Zhu*, Zhiyao He*

E-mail addresses: yunke_zhu@qq.com (Y. Zhu), heyaode@163.com (Z. He)

1.1 **Synthesis of DL-PGNCA**

DL-propargylglycine (5.0 g, 44.2 mmol, 1 eqv) was dissolved in anhydrous THF (60 mL) under Ar₂, followed by addition of α-pinene (32 mL, 4.6 eqv). The mixture was heated to 50 °C, and triphosgene (10.98 g, 37.1 mmol, 0.84 eqv) in THF (15 mL) was added dropwise over 1 h. The reaction proceeded at 50 °C for another 2.5 h until the solution became clear. After solvent removal under reduced pressure distillation, the residue was washed with diethyl ether and stored in dry ether (20 mL) at −20 °C overnight. The precipitate was redissolved in THF (20 mL), filtered, and reprecipitated with diethyl ether. The recrystallization was repeated three times to afford DL-PGNCA as a white crystalline solid.

1.2 **Synthesis of mPEG-NCA**

The block copolymer mPEG-2K-NH₂ (163 mg, 0.815 mmol, 1 eqv) and the DL-PGNCA (800 mg, 5.705 mmol, 7 eqv,) were separately dissolved in anhydrous dimethyl sulfoxide (DMSO) under nitrogen atmosphere after being dried via azeotropic distillation with toluene. The two solutions were then combined and allowed to react at 37 °C for 5 days. The resulting product was purified by dialysis against water for 2 days, followed by lyophilization to afford the final polypeptide product with.

1.3 **Synthesis of ELPM**

The mPEG-NCA (300 mg, 0.0058 mmol, 1 eqv) was dissolved in DMSO (30 mL) under a nitrogen atmosphere. 2-Mercaptoethanol (374 mg, 0.04785 mmol, 82.5 eqv) and the photo-initiator DMPA (80 mg) were added to the solution. The reaction mixture was irradiated with UV light (365 nm) for 4 h under stirring. The product was then purified by dialysis against for 24 h, followed by deionized water for another 24 h, using a dialysis membrane (MWCO: 1 KDa). The final modified copolymer was obtained as a white solid after lyophilization (mPEG-NCA-OH). (*1*)L-BSO (205 mg, 0.924 mmol, 66 eqv.) was activated for 10 minutes with HOBT (125 mg, 0.924 mmol, 66 eqv), HATU (350 mg, 0.924 mmol, 66 eqv), and DIPEA (119mg, 0.924 mmol, 66 eqv). The solution was then added to the PEG-NCA-OH (150 mg, 0.014 mmol, 1eqv) dissolved in DMSO. The crude product was purified by dialysis against a 50% ethanol/water solution using a membrane (MWCO: 1 KDa), yielding the final conjugate (ELPM) as a solid after lyophilization.

Figure S1. Synthetic routes for obtaining ELPM. (A) DL-PGNCA; (B) mPEG-NCA; (C) ELPM.

Figure S2. ^1^H NMR spectrum (400 MHz) of mPEG-NCA in DMSO.

Figure S3. ^1^H NMR spectrum (400 MHz) of ELPM in DMSO.

Figure S4. The TEM image of Es@NPs.

Figure S5. Release of copper ions from EsCu@NPs (n = 3).

Figure S6. L-BSO dose-dependently decreases intracellular GSH levels and enhances the cytotoxic effect of EsCu. (A) Intracellular GSH levels after treatment with different concentrations of L-BSO. (B) Heatmap of cell viability after combined treatment with different concentrations of L-BSO and EsCu. Data are presented as mean ± SD (n = 3)

Figure S7. Western blot showing TXNRD1 expression in 4T1 cells after the indicated treatments.

Figure S8. EsCu@NPs enhanced intracellular ROS accumulation in 4T1 cells. (A) Representative flow cytometry histograms of intracellular ROS levels after different treatments. (B) Quantification of ROS mean fluorescence intensity (MFI). Data are presented as mean ± SD (n = 3).

Figure S9. Effects of different treatments on CRT exposed and HMGB1 released in 4T1 cells.

Figure S10. The CRT exposed in tumor tissues.

Figure S11. The expression of HMGB1 in tumor tissues.

Figure S12. EsCu@NPs promote BMDC maturation in vitro. (A) Representative flow cytometry plots of BMDC maturation. (B) Quantitative analysis of the percentage of mature BMDC. Data are presented as mean ± SD (n = 3).

Figure S13. The infiltration of CD8⁺ T-cells in tumor tissues.

Figure S14. Analysis of intracellular GSH levels in CD8⁺ T cells from peripheral immune organs after different treatments. (A) Relative GSH levels in CD8⁺ T cells isolated from spleens. (B) Relative GSH levels in CD8⁺ T cells isolated from lymph nodes. Data are presented as mean ± SD (n = 3).

Figure S15. Effects of EsCu@NPs on the proportion and exhaustion phenotypes of splenic CD8⁺ T cells. (A) Representative flow cytometry plots of splenic CD8⁺ T cells. (B–C) Representative flow cytometry plots of PD-1⁺LAG-3⁺ and PD-1⁺TIM-3⁺ cells in splenic CD8⁺ T cells. (D–F) Quantitative analysis of the proportion and exhaustion phenotypes of CD8⁺ T cells in each group. Data are presented as mean ± SD (n = 3).

Figure **S16. Exhaustion analysis of tumor-infiltrating CD8⁺ T cells.** (A–C) Representative flow cytometry plots of PD-1^+^LAG-3^+^, PD-1^+^TIM-3^+^, and PD-1^+^ expression in tumor-infiltrating CD8⁺ T cells. (D–F) Quantification of PD-1⁺LAG-3⁺, PD-1⁺TIM-3⁺, and PD-1⁺CD8⁺ T cells (n = 3).

Figure S17. Gating strategies for analysis DC cells by FCM.

Figure S18. Gating strategies for analysis immune cells by FCM.

Figure S19. Ex vivo biodistribution of ICG@NPs at 48 h in major organs and tumor, highlighting tumor accumulation.

Figure S20. EsCu@NPs enhances Cu delivery and prolongs blood circulation in vivo. (A) Comparison of Cu content in tumor among different formulations. (B) Blood circulation profiles of EsCu and EsCu@NPs in mice. Data are presented as mean ± SD (n = 3).

Figure S21. Copper ion content in mouse tumor tissues after different treatments. Data are mean ± SD (n = 3).

Figure S22. Blood test results. Blood routine examinations (A) and serum biochemistry data (B) of mice. Data are mean ± SD (n = 3).

Figure S23. Histological analysis of major organs. H&E staining of heart, liver, lung and kidney from 4T1 tumor-bearing mice.

Figure S24. analysis of major organs coefficient after different treatments.

Figure S25. Hemolysis rates of RBCs after incubation with different concentrations of EsCu@NPs (0.0625–2 mg/mL). PBS and Triton X-100 were used as the negative and positive controls, respectively (n = 3).

Figure S26. Western blot analysis of DLAT aggregation, FDX1, and LIAS in tumor tissues after different treatments.

Figure S27. EsCu@NPs regulate cuproptosis-associated markers in tumor tissues. (A) Representative immunohistochemical staining images of DLAT, FDX1, and LIAS in tumor tissues after different treatment. (B–D) Quantitative analysis of DLAT, FDX1, and LIAS-positive signals in tumor.

Figure S28. Percentage of necrotic area to total tumor area following different treatments (n=3).

Figure S29. Photographs of excised tumors at the study endpoint (n = 6 mice).

Figure S30. Migration quantification at 24 h (A) and 48 h (B). Data are mean ± SD (n = 3).

Figure S31. Blood test results. Blood routine examinations (A) and serum biochemistry data (B) of mice when reached the endpoint of tumor size. Data are mean ± SD (n = 3).

Figure S32. Histological analysis of major organs. H&E staining of heart, liver, lung and kidney from 4T1 tumor-bearing mice.

Figure S33. Organ index analysis of major organs after different treatments.

Figure S34. Percentage of necrotic area to total tumor area following different treatments (n=3).

Figure S35. Percentage of Ki-67 positive signals following different treatments (n=3).

Figure S36. CD8^+^ T cells infiltration following different treatments (n = 3).
